# Gastroprotective Effects of *Salvia plebeia* via Antioxidant and MAPK/NF-κB-Mediated Anti-Inflammatory Mechanisms in Ethanol/HCl-Induced Gastric Injury

**DOI:** 10.3390/ijms27104358

**Published:** 2026-05-14

**Authors:** Yun-seong Lee, Sunju So, Hyun-A Lee

**Affiliations:** 1 Nain Healthcare Co., Ltd., 3, Seodong-ro 20-gil, Iksan 54613, Republic of Korea; iyoonseong@daum.net; 2 College of Korean Medicine, Woosuk University, Jeonju 55338, Republic of Korea; thtnswn1016@naver.com; 3 Center for Animal Resources Development, Wonkwang University, Iksan 54538, Republic of Korea

**Keywords:** *Salvia plebeia*, gastritis, antioxidant, anti-inflammatory, cytokines

## Abstract

This study investigated the gastroprotective effects of *Salvia plebeia* extract (SPE) against acute gastric mucosal injury induced by 150 mM HCl/60% ethanol in rats and explored its antioxidant and anti-inflammatory mechanisms. SPE exhibited strong in vitro antioxidant activity, with DPPH and ABTS radical scavenging rates of 86.2 ± 2.4% and 89.1 ± 1.9%, respectively, along with a high total polyphenol content (96.4 ± 3.1 mg gallic acid equivalents/g extract). In lipopolysaccharide (LPS)-stimulated RAW 264.7 macrophages, SPE attenuated LPS-induced inflammatory signaling, as evidenced by reduced TLR4 and JNK expression and restoration of IκBα levels. In vivo, oral administration of SPE (100 or 300 mg/kg) 1 h prior to HCl/ethanol challenge significantly reduced gastric lesion area and improved histopathological damage compared with the HCl/ethanol-treated control group. SPE also increased gastric pH, reduced gastric juice volume, decreased serum levels of TNF-α and IL-6, and downregulated gastric mucosal mRNA expression of Nos2 and Ptgs2. Immunohistochemical analysis further showed that SPE attenuated NF-κB p65 immunoreactivity in gastric tissues. Collectively, these findings suggest that SPE exerts gastroprotective effects through antioxidant activity and suppression of inflammatory responses associated with the MAPK/NF-κB pathway in acute HCl/ethanol-induced gastric injury.

## 1. Introduction

Gastric mucosal injury is a multifactorial gastrointestinal disorder that develops when aggressive luminal factors overwhelm the endogenous defensive mechanisms that normally preserve epithelial integrity [1]. The gastric mucosa is protected by a coordinated system consisting of surface mucus, bicarbonate secretion, prostaglandins, antioxidant defenses, epithelial restitution, and adequate mucosal blood flow [2]. When this balance collapses, acute gastric lesions characterized by hemorrhage, edema, epithelial exfoliation, and erosions can rapidly occur [3]. This finding emphasizes that acute gastritis results from an imbalance between mucosal aggressors and defensive mechanisms, with oxidative stress and inflammatory cascades playing central roles in lesion progression. Among experimental ulcer models, HCl/ethanol-induced gastric injury is widely used because it produces rapid and reproducible gastric mucosal damage and closely mimics acute corrosive lesions [4]. Ethanol disrupts the mucus barrier, causes microvascular stasis, and increases epithelial permeability, thereby facilitating penetration of hydrogen ions into the mucosa [5]. Acid co-administration intensifies this process, accelerating necrosis, hemorrhagic band formation, and gross ulcerative damage [6]. In rodent models, HCl/ethanol challenge consistently produces clear endoscopic, gross, biochemical, and histopathological changes, which makes it highly suitable for evaluating natural gastroprotective agents [7].

Oxidative stress is a major pathogenic event in ethanol and HCl/ethanol-induced gastric injury. Ethanol has been shown to act as a direct oxidative stressor in gastric epithelial cells by inducing intracellular superoxide generation and mitochondrial dysfunction [8]. Excessive ROS production promotes lipid peroxidation, membrane destabilization, DNA damage, and depletion of endogenous antioxidants such as glutathione, catalase, and superoxide dismutase [9]. In the HCl/ethanol model, restoration of antioxidant enzyme activity and reduction of oxidative biomarkers such as ROS, MDA, and 8-OHdG are closely associated with attenuation of gastric lesion severity [10]. ROS overproduction and oxidative damage are recognized as core mechanisms in gastritis and support the use of antioxidant phytochemicals as potential therapeutic candidates.

Inflammation further amplifies oxidative mucosal injury and contributes to the transition from superficial epithelial damage to deeper tissue destruction [11]. Ethanol-related gastric injury is accompanied by increased levels of pro-inflammatory mediators, including TNF-α, IL-6, IL-1β, iNOS and COX-2, together with enhanced neutrophil infiltration and myeloperoxidase activity [12]. Studies using phenolic or flavonoid gastroprotective agents have repeatedly shown that sup-pression of these inflammatory mediators is paralleled by improvement of histological architecture and reduction of gross lesion area [13]. Accordingly, phenolic compounds and flavonoids may ameliorate gastritis through antioxidant, anti-inflammatory, and cytoprotective mechanisms, which is directly relevant to the present study design. At the signaling level, the MAPK/NF-κB axis is one of the most important molecular hubs linking oxidative stress to inflammatory gene transcription in gastric injury [14]. NF-κB activation promotes transcription of cytokines and inflammatory enzymes such as TNF-α, IL-6, iNOS and COX-2, thereby perpetuating epithelial injury and delaying mucosal recovery [15]. JNK and related MAPK pathways are activated by oxidative and inflammatory stimuli and contribute to amplification of gastric inflammatory responses [16]. In addition, up-stream pattern-recognition signaling, including TLR4-associated pathways in macrophages, has been recognized as a critical initiator of inflammatory cytokine production and NF-κB activation under injurious conditions. Accordingly, botanical extracts capable of inhibiting TLR4/JNK/IκBα/NF-κB signaling are mechanistically attractive candidates for gastroprotection [17].

*Salvia plebeia* is a medicinal herb widely distributed in East Asia and traditionally used for the treatment of inflammatory disorders, cough, and bronchitis [18]. Modern phytochemical studies have shown that *S. plebeia* contains diverse phenolic acids and flavonoids, including rosmarinic acid, caffeic acid, luteolin, homoplantaginin, hispidulin, and related constituents [19]. A broad pharmacological review further concluded that *S. plebeia* possesses antioxidant, anti-inflammatory, antimicrobial, antiasthmatic, hepatoprotective, and other bioactivities relevant to inflammatory tissue injury [20]. This phytochemical profile is highly compatible with the pathological themes emphasized in the uploaded gastritis.pdf, particularly the importance of phenolics and flavonoids in oxidative and inflammatory gastric damage. Several prior studies support the anti-inflammatory potential of *S. plebeia* at both the extract and constituent levels. Jung et al. demonstrated that an ethanol extract of *S. plebeia* exerted significant anti-inflammatory, anti-angiogenic and anti-nociceptive activities in experimental systems [21]. Jang et al. reported that *S. plebeia* extract ameliorated inflammatory responses in RAW 264.7 and BEAS-2B cells and improved inflammatory histopathology in an ovalbumin-induced mouse model [22]. Kim et al. further showed that nepetoidin B isolated from *S. plebeia* inhibited inflammatory responses in macrophages by modulating NF-κB and Nrf2/HO-1 signaling pathways [23]. These findings strongly suggest that *S. plebeia* contains active components capable of regulating the same redox-sensitive inflammatory networks implicated in acute gastric mucosal injury. Importantly, Nugroho et al. reported that *S. plebeia* extract and its ethyl acetate fraction were effective in both HCl/EtOH-induced and indomethacin/bethanechol-induced gastric lesion assays, and rosmarinic acid was identified as a major active polyphenol [24]. Independently, rosmarinic acid itself has been shown to prevent gastric ulcers through cytoprotective, antioxidant, sulfhydryl-reinforcing, and immunomodula-tory mechanisms [25]. Heidari et al. also reported that rosmarinic acid protected against ethanol-induced gastritis in male rats, supporting its relevance as a gastric protective phytochemical source cited within the uploaded material. Together, these reports provide a strong biochemical and pharmacological rationale for investigating *Salvia plebeia* extract (SPE) in acute gastric injury. Based on these observations, SPE can be hypothesized to exert gastroprotective activity through dual antioxidant and anti-inflammatory mechanisms.

In the present study, SPE showed strong DPPH and ABTS radical scavenging activities and a high total polyphenol content, suggesting notable antioxidant potential. In vivo, SPE reduced gastric lesion area, improved histopathological damage, increased gastric pH, and reduced gastric juice volume in rats with HCl/ethanol-induced gastric injury, while also lowering serum TNF-α and IL-6 levels and suppressing gastric mucosal expression of Nos2 and Ptgs2. In addition, SPE modulated TLR4/JNK/IκBα-associated inflammatory signaling in LPS-stimulated RAW 264.7 macrophages and attenuated NF-κB p65 immunoreactivity in gastric tissues. Therefore, the aim of this study was to evaluate the gastroprotective effects of SPE in an HCl/ethanol-induced rat model and to examine whether its protective actions are associated with antioxidant activity and suppression of inflammatory signaling related to the MAPK/NF-κB pathway.

## 2. Results

### 2.1. Antioxidant Capacity and Polyphenol Content

**Figure 1:**
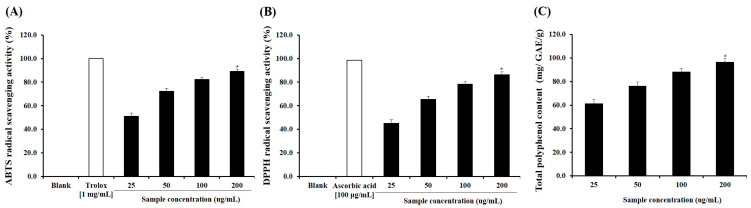
Antioxidant capacity and total polyphenol content of Salvia plebeia extract (SPE). (**A**) DPPH radical scavenging activity, (**B**) ABTS radical scavenging activity, (**C**) Total polyphenol content (TPC). * *p* < 0.05 vs. control. Values are expressed as mean ± SD (*n* = 3).

The antioxidant properties of SPE were evaluated using DPPH and ABTS radical scavenging assays, along with determination of total polyphenol content (TPC). SPE exhibited strong antioxidant activity, with DPPH and ABTS radical scavenging rates of 86.2 ± 2.4% and 89.1 ± 1.9%, respectively (Figure 1A, B and C). In addition, the TPC of SPE was measured as 96.4 ± 3.1 mg gallic acid equivalents (GAE)/g extract, indicating a high level of phenolic compounds. These results demonstrate that SPE possesses substantial free radical scavenging capacity, which may contribute to its biological activity.

### 2.2. HPLC Chromatographic Profiling of SPE

**Figure 2:**
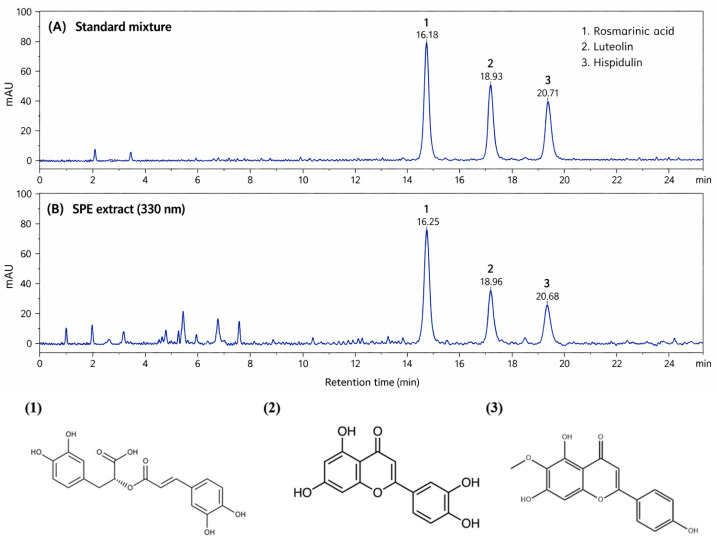
HPLC chromatographic profiles of SPE and reference standards. (**A**) Standard mixture containing rosmarinic acid (1), luteolin (2), and hispidulin (3). (**B**) SPE extract detected at 330 nm. Peaks were identified by comparison of retention times with those of the reference compounds.

The phytochemical composition of SPE was analyzed by HPLC–PDA, and representative chromatograms are shown in Figure 2. As illustrated in Figure 2A, the standard mixture exhibited three well-resolved peaks corresponding to rosmarinic acid, luteolin, and hispidulin. These compounds were eluted at retention times of approximately 16.1–16.3 min, 18.8–19.0 min, and 20.6–20.8 min, respectively. The chromatographic profile of SPE (Figure 2B) revealed multiple peaks, among which three major peaks were clearly observed at retention times comparable to those of the reference standards. These peaks were identified as rosmarinic acid (Peak 1), luteolin (Peak 2), and hispidulin (Peak 3). Notably, the peak corresponding to rosmarinic acid showed the highest intensity among the detected compounds, indicating that it is the predominant phenolic constituent in SPE. In addition, several minor peaks were observed in the earlier retention time region, suggesting the presence of other polar phytochemicals. These chromatographic results confirm that SPE contains characteristic phenolic and flavonoid compounds commonly reported in SPE. In addition to rosmarinic acid, luteolin, and hispidulin, several unidentified minor peaks were observed in the chromatographic profile of SPE, suggesting the presence of additional polar phenolic compounds and flavonoid derivatives. These minor constituents may also contribute synergistically to the antioxidant and anti-inflammatory activities of SPE.

### 2.3. Effects of SPE on MAPK/NF-κB Signaling in LPS-Stimulated Macrophages

**Figure 3:**
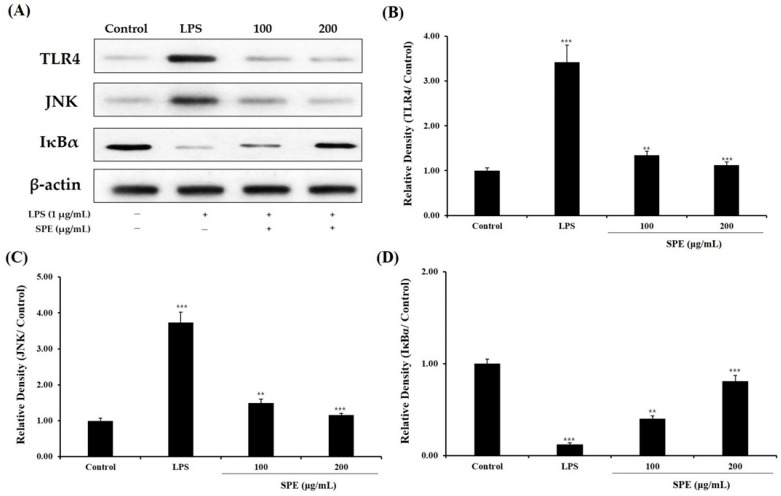
Effects of SPE on LPS-induced TLR4-mediated MAPK and NF-κB signaling pathways in RAW 264.7 cells. (**A**) Representative western blot images showing the expression levels of TLR4, JNK, and IκBα in RAW 264.7 cells treated with LPS and SPE. β-actin was used as the loading control. (**B**) Relative protein expression level of TLR4. (**C**) Relative protein expression level of JNK. (**D**) Relative protein expression level of IκBα. Statistical significance was determined by one-way ANOVA followed by Tukey’s post hoc test. *** *p* < 0.001 vs. control group; ** *p* < 0.01 and *** *p* < 0.001 vs. LPS-treated group.

The effects of SPE on LPS-induced inflammatory signaling-related proteins were investigated in RAW 264.7 macrophages by Western blot analysis (Figure 3A). As shown in Figure 3B–D, LPS stimulation significantly increased the expression levels of TLR4 and JNK compared with the control group, whereas the expression level of IκBα was markedly decreased, indicating activation of inflammatory signaling. Treatment with SPE attenuated these LPS-induced alterations in a concentration-dependent manner. Specifically, SPE at 100 and 200 μg/mL reduced the elevated expression levels of TLR4 and JNK compared with the LPS-treated group. In addition, SPE restored the reduced IκBα level, with the 200 μg/mL treatment showing the most pronounced effect. These findings suggest that SPE modulates LPS-induced inflammatory signaling associated with the MAPK/NF-κB pathway in macrophages.

### 2.4. Effect of SPE on Clinical Symptom Scores

**Figure 4:**
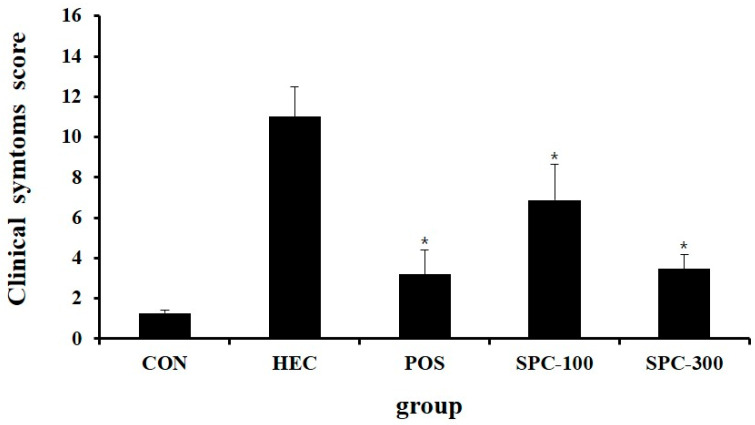
Effects of mixture of SPE on the clinical symptom score in a HCl/ethanol-induced gastritis animal model. CON; Normal control, HEC; 150 mM HCl-60% ethanol + D.W, POS; 150 mM HCl-60% ethanol + Ranitidine 30 mg/kg, SPC-100; 150 mM HCl-60% ethanol + *Salvia plebeia* extract 100 mg/kg, SPC-300; 150 mM HCl-60% ethanol + *Salvia plebeia* extract 300 mg/kg. Values are expressed as mean ± SD (*n* = 5). * *p* < 0.05 vs. HEC.

Given the limited number of in vivo studies investigating the gastroprotective effects of *Salvia plebeia*, the present study prioritizes physiological and histopathological outcomes in the HCl/ethanol-induced gastric injury model. In vivo parameters, including gastric lesion area, inhibition ratio, gastric secretion, cytokine levels, and tissue morphology, were comprehensively evaluated to establish the translational relevance of SPE. Clinical symptoms were evaluated following administration of SPE in rats with HCl/ethanol-induced gastric injury. The normal control group exhibited a clinical symptom score of 1.27 ± 0.15, reflecting normal behaviors such as locomotion, grooming, and feeding. In contrast, the HCl/ethanol-treated control group (HEC) showed a markedly elevated score of 11.00 ± 1.49 indicating severe impairment characterized by minimal movement and weak responsiveness to external stimuli. Compared with the HEC group, the positive control group treated with ranitidine showed a significantly reduced score of 3.20 ± 1.19 (* *p* < 0.05 vs. HEC). Similarly, SPE treatment improved clinical symptoms in a dose-dependent manner, with scores of 6.87 ± 1.77 in the SPC-100 group and 3.47 ± 0.73 in the SPC-300 group (* *p* < 0.05 vs. HEC). These results indicate that SPE alleviated the severity of clinical symptoms induced by HCl/ethanol treatment (Figure 4).

### 2.5. Effect of SPE on Gastric Lesion Area and Inhibition Ratio

**Figure 5:**
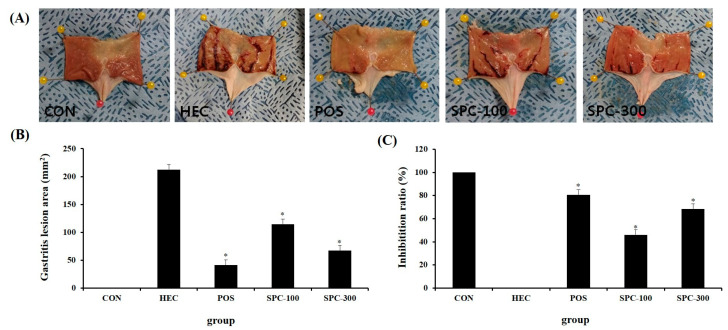
Effects of SPE on gastric lesion area and inhibition ratio in HCl/ethanol-induced gastric injury in rats. (**A**) Representative images of gastric mucosal lesions in each experimental group. (**B**) Quantitative analysis of gastritis lesion area. (**C**) Inhibition ratio of gastric mucosal injury in each treatment group. CON; Normal control, HEC; 150 mM HCl-60% ethanol + D.W, POS; 150 mM HCl-60% ethanol + Ranitidine 30 mg/kg, SPC-100; 150 mM HCl-60% ethanol + *Salvia plebeia* extract 100 mg/kg, SPC-300; 150 mM HCl-60% ethanol + *Salvia plebeia* extract 300 mg/kg. Gross gastric lesion areas were measured after HCl/ethanol administration. Inhibition ratio (%) was calculated relative to the HEC group. Values are expressed as mean ± SD (*n* = 5). * *p* < 0.05 vs. HEC.

Gross gastric lesion areas were measured to evaluate the protective effect of SPE against HCl/ethanol-induced gastric injury. No visible lesions were observed in the normal control group (CON; 0.0 ± 0.0 mm^2^), whereas the HCl/ethanol-treated control group (HEC) exhibited severe gastric mucosal damage with a lesion area of 212.6 ± 9.3 mm^2^ characterized by extensive hemorrhagic streaks and mucosal disruption (Figure 5A,B). In contrast, the ranitidine-treated group (POS) showed a significantly reduced lesion area of 41.0 ± 9.3 mm^2^ (* *p* < 0.05 vs. HEC). Similarly, SPE treatment markedly reduced gastric lesion areas to 114.6 ± 9.2 mm^2^ in the SPC-100 group and 67.2 ± 9.6 mm^2^ in the SPC-300 group (* *p* < 0.05 vs. HEC), indicating a dose-dependent protective effect. To further quantify the protective effect, inhibition ratios were calculated relative to the HEC group. The POS group exhibited an inhibition rate of 80.7%, while the SPC-100 and SPC-300 groups showed inhibition rates of 46.1% and 68.4%, respectively (Figure 5C). These results demonstrate that SPE significantly attenuated HCl/ethanol-induced gastric mucosal injury in a dose-dependent manner.

### 2.6. Effect of SPE on Gastric Juice pH and Volume

**Figure 6:**
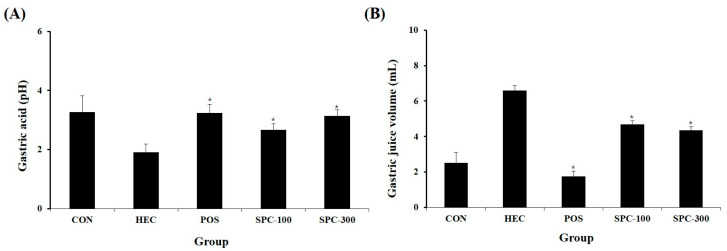
Effects of SPE on (**A**) gastric juice pH and (**B**) gastric juice volume in HCl/ethanol-induced gastric injury in rats. CON; Normal control, HEC; 150 mM HCl-60% ethanol + D.W, POS; 150 mM HCl-60% ethanol + Ranitidine 30 mg/kg, SPC-100; 150 mM HCl-60% ethanol + *Salvia plebeia* extract 100 mg/kg, SPC-300; 150 mM HCl-60% ethanol + *Salvia plebeia* extract 300 mg/kg. Values are expressed as mean ± SD (*n* = 5). * *p* < 0.05 vs. HEC.

The effects of SPE on gastric secretion were evaluated by measuring gastric juice pH and volume (Figure 6A,B). The HEC group showed a significantly lower gastric pH (1.91 ± 0.28) compared with the normal control group (3.26 ± 0.56). SPE treatment significantly increased gastric pH in a dose-dependent manner reaching 2.67 ± 0.21 (SPC-100) and 3.13 ± 0.21 (SPC-300) (* *p* < 0.05 vs. HEC). The SPC-300 group showed values comparable to those of the ranitidine-treated group (3.23 ± 0.31). Regarding gastric juice volume, the HEC group exhibited a marked increase (6.59 ± 1.04 mL) compared with the normal control group (2.53 ± 0.75 mL). SPE treatment significantly reduced gastric secretion to 4.69 ± 0.34 mL (SPC-100) and 4.35 ± 0.43 mL (SPC-300) (* *p* < 0.05 vs. HEC). These findings indicate that SPE attenuated HCl/ethanol-induced gastric hypersecretion and acidity.

### 2.7. Effect of SPE on Serum Inflammatory Cytokine Levels

**Figure 7:**
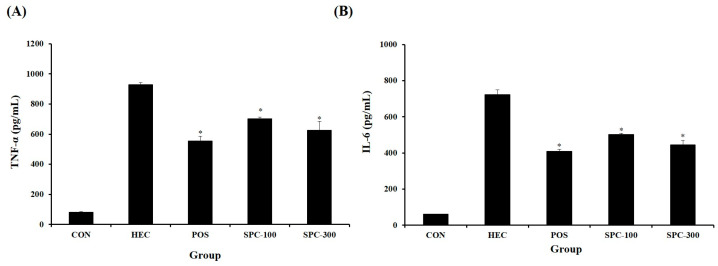
Effects of SPE on serum (**A**) TNF-α and (**B**) IL-6 levels in HCl/ethanol-induced gastric injury in rats. CON; Normal control, HEC; 150 mM HCl-60% ethanol + D.W, POS; 150 mM HCl-60% ethanol + Ranitidine 30 mg/kg, SPC-100; 150 mM HCl-60% ethanol + *Salvia plebeia* extract 100 mg/kg, SPC-300; 150 mM HCl-60% ethanol + *Salvia plebeia* extract 300 mg/kg. Values are expressed as mean ± SD (*n* = 5). * *p* < 0.05 vs. HEC.

Serum TNF-α and IL-6 levels were measured to evaluate the anti-inflammatory effect of SPE. HCl/ethanol treatment significantly increased TNF-α and IL-6 levels compared with the normal control group. SPE administration significantly reduced TNF-α and IL-6 levels compared with the HEC group (* *p* < 0.05 vs. HEC), indicating suppression of systemic inflammatory responses (Figure 7A,B).

### 2.8. Effects of SPE on Pro-Inflammatory Gene Expression in Gastric Tissues

**Figure 8:**
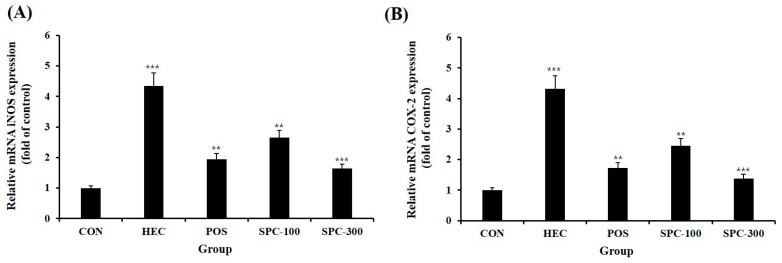
Effects of SPE on (**A**) iNOS and (**B**) COX-2 mRNA expression in gastric tissues of HCl/ethanol-induced gastric injury rats. Data are expressed as mean ± SD (*n* = 5). ** *p* < 0.01 and *** *p* < 0.001 compared with the CON group.

To further investigate the anti-inflammatory effects of SPE in vivo, the mRNA expression levels of pro-inflammatory mediators were analyzed in gastric tissues using quantitative real-time PCR (Figure 8A,B). HCl/ethanol administration markedly upregulated the mRNA expression levels of inducible nitric oxide synthase (iNOS; Nos2) and cyclooxygenase-2 (COX-2; Ptgs2) compared with the normal control group (*p* < 0.001), indicating a pronounced inflammatory response in gastric tissues. Oral administration of SPE significantly attenuated the elevated expression of these genes. Both SPC-100 and SPC-300 groups showed significant reductions in iNOS and COX-2 mRNA levels compared with the HCl/ethanol-treated group (*p* < 0.01 or *p* < 0.001). Notably, the SPC-300 group exhibited the lowest expression levels among the SPE-treated groups, demonstrating a stronger inhibitory effect than SPC-100. However, the positive control (POS) group showed a more pronounced suppression of iNOS and COX-2 expression than the SPC-100 group, indicating that the anti-inflammatory effect of SPE, while dose-dependent, did not fully surpass that of the standard treatment at the tested doses. These results indicate that SPE effectively suppresses inflammatory responses at the transcriptional level in vivo, which is consistent with the observed reductions in serum pro-inflammatory cytokines (TNF-α and IL-6) and the improvement in gastric mucosal damage.

### 2.9. Histopathological Evaluation of Gastric Tissues

**Figure 9:**
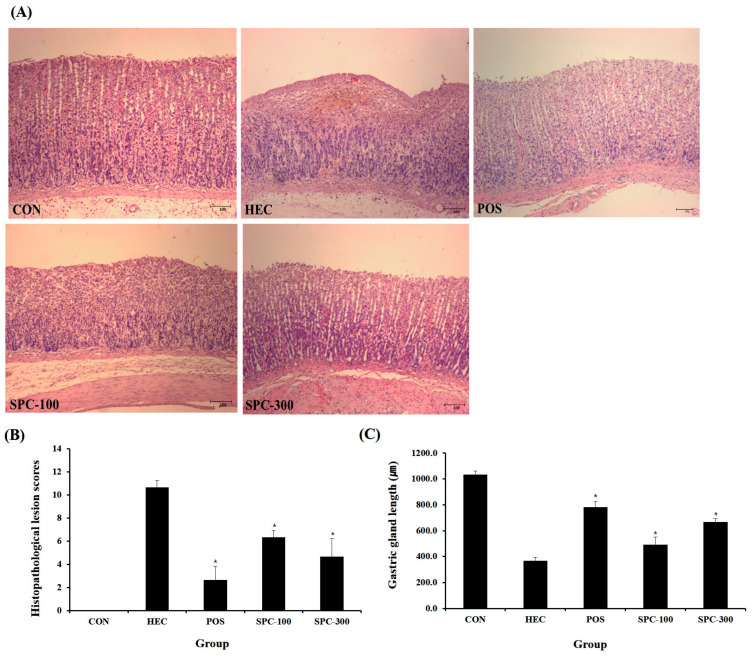
Effects of SPE on histopathological changes and gastric gland length in an HCl/ethanol-induced gastritis rat model. (**A**) Representative histological images of gastric tissues stained with H&E in each experimental group. (**B**) Quantitative analysis of histopathological lesion scores. (**C**) Quantitative analysis of gastric gland length. CON; Normal control, HEC; 150 mM HCl-60% ethanol + D.W, POS; 150 mM HCl-60% ethanol + Ranitidine 30 mg/kg, SPC-100; 150 mM HCl-60% ethanol + *Salvia plebeia* extract 100 mg/kg, SPC-300; 150 mM HCl-60% ethanol + *Salvia plebeia* extract 300 mg/kg. Sections were counterstained with hematoxylin & Eosin. Scale bar = 100 μm. Magnification: 200×. Values are expressed as mean ± SD (*n* = 5). * *p* < 0.05 vs. HEC.

Histopathological examination was performed to evaluate the protective effects of SPE against HCl/ethanol-induced gastric mucosal injury (Figure 9A–C). The HCl/ethanol-treated group (HEC) exhibited severe gastric damage characterized by marked epithelial disruption, hemorrhage, and mucosal erosion compared with the normal control group (CON). These histological alterations were further supported by a significant increase in histopathological lesion scores and a marked reduction in gastric gland length (*p* < 0.001 vs. CON). In contrast, treatment with SPE significantly attenuated gastric mucosal damage. Both SPC-100 and SPC-300 groups showed reduced epithelial disruption and inflammatory infiltration compared with the HEC group, accompanied by a significant decrease in lesion scores and partial restoration of gastric gland length (*p* < 0.05 or *p* < 0.01 vs. HEC). Notably, the SPC-300 group exhibited greater protective effects than SPC-100, indicating a dose-dependent improvement. However, the positive control (POS, ranitidine-treated group) showed the most pronounced protective effect, with the lowest lesion scores and the most substantial recovery of gastric gland length among all treatment groups. Although SPC-300 markedly improved gastric mucosal integrity, its protective effect did not fully reach the level observed in the POS group. These histopathological findings were consistent with the overall anti-inflammatory effects observed in SPE-treated groups.

### 2.10. Effects of SPE on NF-κB Activation in Gastric Tissues (IHC Analysis)

**Figure 10:**
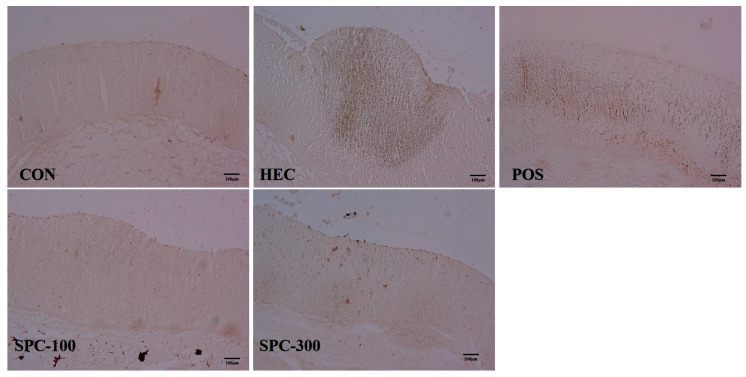
Representative IHC staining of NF-κB p65 in gastric tissues from an HCl/ethanol-induced gastritis model. CON; Normal control, HEC; 150 mM HCl-60% ethanol + D.W, POS; 150 mM HCl-60% ethanol + Ranitidine 30 mg/kg, SPC-100; 150 mM HCl-60% ethanol + *Salvia plebeia* extract 100 mg/kg, SPC-300; 150 mM HCl-60% ethanol + *Salvia plebeia* extract 300 mg/kg. Sections were counterstained with hematoxylin & Eosin. Scale bar = 100 μm. Magnification: 200.

Immunohistochemical analysis was performed to evaluate the effect of SPE on NF-κB activation in gastric tissues (Figure 10). NF-κB expression was minimal in the control group, with only weak and localized staining predominantly observed in the cytoplasm of gastric epithelial cells. In contrast, the HCl/ethanol-treated group (HEC) exhibited markedly increased NF-κB expression, characterized by strong immunoreactivity and prominent nuclear localization in gastric mucosal cells, indicating activation of NF-κB signaling. Treatment with SPE significantly attenuated NF-κB expression compared with the HEC group. In particular, the SPC-100 group showed a moderate reduction in NF-κB immunoreactivity, whereas the SPC-300 group exhibited a pronounced decrease, with staining patterns approaching those of the control group. Similarly, the positive control group (ranitidine) also demonstrated reduced NF-κB expression. Overall, SPE reduced NF-κB p65 immunoreactivity in gastric tissues in a dose-dependent manner, suggesting inhibition of NF-κB-associated inflammatory signaling in vivo.

## 3. Discussion

The present study demonstrates that *Salvia plebeia* extract (SPE) exerts significant gastroprotective effects against HCl/ethanol-induced gastric mucosal injury, which are closely associated with attenuation of oxidative stress and suppression of inflammatory signaling pathways [26,27]. Ethanol/HCl-induced gastric injury is closely associated with transition metal-catalyzed oxidative reactions, including Fe- and Cu-mediated Fenton-type hydroxyl radical generation [28,29]. These metal-dependent oxidative processes can accelerate lipid peroxidation, epithelial disruption, and inflammatory amplification in gastric tissues [30]. Although direct analyses of Fe (II/III)- and Cu (I/II)-associated ROS generation were not performed in the present study, such oxidative mechanisms may partially contribute to the gastric injury microenvironment observed in the HCl/ethanol model. Because direct oxidative stress biomarkers such as MDA, GSH, SOD, and catalase activity were not measured in vivo, the present findings should be interpreted as evidence of antioxidant-associated protective activity rather than definitive proof of oxidative stress reduction. SPE exhibited strong antioxidant activity, as evidenced by high radical scavenging capacity and polyphenol content, which are known to play critical roles in protecting gastric mucosa from oxidative damage [31]. Previous studies have shown that polyphenol-rich plant extracts effectively ameliorate ethanol-induced gastric injury through antioxidant and anti-inflammatory mechanisms [32]. The HPLC chromatographic analysis of SPE demonstrated the presence of key phenolic and flavonoid compounds, including rosmarinic acid, luteolin, and hispidulin. Among the identified compounds, rosmarinic acid is likely a major contributor to the antioxidant activity of SPE, whereas luteolin and hispidulin may contribute predominantly to anti-inflammatory signaling modulation through regulation of MAPK/NF-κB-associated pathways, which is consistent with previous reports on *Salvia plebeia* extracts and related Lamiaceae plants [33]. Rosmarinic acid is widely recognized for its potent antioxidant activity, particularly its ability to scavenge reactive oxygen species and inhibit lipid peroxidation [34]. These findings are consistent with previous comprehensive reviews of *Salvia plebeia*, which have reported that its rich phenolic and flavonoid composition contributes to diverse pharmacological activities, including antioxidant and anti-inflammatory effects. In particular, compounds such as rosmarinic acid and luteolin are known to modulate redox-sensitive signaling pathways, supporting the mechanistic basis of the gastroprotective effects observed in the present study. Polyphenolic constituents identified in SPE, including rosmarinic acid, luteolin, and related flavonoids, may additionally exert indirect antioxidant-associated effects through transition metal-chelating activity [35,36]. Because hydroxyl-containing phenolic structures can interact with Fe and Cu ions, these compounds may partially attenuate Fenton-type hydroxyl radical generation and oxidative tissue injury [37]. However, direct metal-chelating activity was not experimentally evaluated in the present study and therefore requires further investigation. In addition, flavonoids such as luteolin and hispidulin have been reported to exert anti-inflammatory effects through modulation of key signaling pathways, including MAPK and NF-κB pathways [38,39]. Therefore, the presence of these bioactive constituents provides a plausible chemical basis for the gastroprotective effects observed in the present study. It is likely that the biological activity of SPE is mediated, at least in part, through the synergistic actions of these phenolic and flavonoid compounds. The antioxidant activity evaluated in the present study was primarily based on chemical radical scavenging assays rather than direct intracellular ROS measurements. Therefore, further studies using ROS-sensitive fluorescence probes or oxidative biomarker analyses are necessary to clarify the direct redox-modulating effects of SPE. In addition, the term antioxidant effect was interpreted with biochemical specificity in the present study. SPE exhibited potent radical scavenging activity in DPPH and ABTS assays, which primarily reflect electron transfer-based free radical scavenging capacity rather than comprehensive antioxidant mechanisms [40]. Moreover, oxidative stress is closely associated with inflammatory signaling pathways, including NF-κB and MAPK activation, while polyphenolic compounds are known to modulate redox-sensitive inflammatory responses [41,42]. Therefore, the observed effects of SPE may be associated with radical scavenging activity and oxidative stress-associated inflammatory modulation mediated by polyphenol-related redox regulation. Furthermore, the chromatographic profile obtained in this study supports the chemical consistency of SPE, reinforcing its reproducibility and potential as a functional phytochemical resource for the prevention or treatment of gastric mucosal injury. Histopathological analysis further confirmed that SPE preserved gastric mucosal integrity by reducing epithelial damage, edema, and inflammatory cell infiltration. These findings are consistent with previous reports demonstrating that natural compounds can attenuate gastric mucosal injury by maintaining epithelial structure and suppressing inflammatory progression [43]. Ethanol-induced gastric damage is characterized by rapid epithelial disruption and subsequent amplification of inflammatory responses, which can be mitigated by antioxidant-rich interventions [44]. Recent studies have demonstrated that gastroprotection by plant-derived phenolics involves integrated mechanisms combining antioxidant defense, inflammatory suppression, and regulation of upstream pattern-recognition signaling pathways. In particular, TLR4-mediated activation of MAPK and NF-κB signaling represents a critical molecular axis linking oxidative stress to inflammatory gene expression in gastric injury. Therefore, beyond conventional antioxidant evaluation, the present study further investigates the upstream regulatory mechanisms of SPE and integrates these findings with in vivo physiological and histopathological outcomes to provide a more comprehensive mechanistic framework. At the molecular level, SPE modulated inflammatory signaling in LPS-stimulated RAW 264.7 macrophages by reducing the expression levels of TLR4 and JNK and restoring IκBα levels, indicating suppression of upstream MAPK/NF-κB-associated signaling [45]. Cytotoxicity assays were not conducted in parallel, the possibility that high concentrations of SPE may partially influence cellular viability cannot be completely excluded. Also, phosphorylated MAPK proteins such as p-ERK and p-JNK were not evaluated in the present study, the precise effects of SPE on MAPK activation status remain to be further elucidated. The present findings should be interpreted as evidence of pathway-associated inflammatory modulation rather than direct mechanistic inhibition of MAPK/NF-κB signaling. Activation of TLR4-mediated signaling cascades is a well-established mechanism leading to NF-κB activation and subsequent transcription of pro-inflammatory mediators [46]. Therefore, the observed suppression of these signaling molecules suggests that SPE effectively blocks inflammatory signal transduction at an early stage. Consistent with the in vitro findings, in vivo results demonstrated that SPE reduced serum levels of TNF-α and IL-6 and downregulated gastric expression of iNOS and COX-2, indicating suppression of both systemic and local inflammatory responses [47]. However, changes in mRNA expression do not necessarily indicate direct inhibition of protein expression or enzymatic activity. Therefore, the present findings should be interpreted as evidence of transcriptional modulation rather than definitive functional inhibition, and further protein-level analyses, including Western blotting and enzymatic activity assays, are required to confirm these effects. These mediators are key contributors to gastric mucosal injury, as they promote nitric oxide overproduction, inflammatory amplification, and tissue damage [48]. The coordinated inhibition of cytokines and inflammatory enzymes suggests that SPE exerts multi-target anti-inflammatory effects. Immunohistochemical analysis further revealed that SPE significantly attenuated NF-κB p65 immunoreactivity in gastric tissues, supporting the inhibition of NF-κB-mediated inflammatory activation in vivo [49]. NF-κB is a central transcription factor regulating inflammatory responses and is activated in various gastric injury models [50]. The reduction of NF-κB activation observed in this study is consistent with the improvements in histopathological and biochemical parameters. While several studies have reported the antioxidant and anti-inflammatory effects of *Salvia plebeia* in cellular systems, in vivo evidence supporting its gastroprotective efficacy remains limited. Therefore, the present findings provide important physiological validation by demonstrating that SPE significantly improves gastric mucosal integrity and suppresses inflammatory responses in an established animal model. Although statistically significant protective effects were observed, the relatively small sample size represents a limitation of the study, and larger-scale studies with formal power calculations are required to confirm reproducibility and statistical robustness.

Collectively, these findings suggest that SPE exerts gastroprotective effects through dual mechanisms involving antioxidant activity and inhibition of MAPK/NF-κB signaling pathways [51]. This integrated mode of action is particularly relevant in HCl/ethanol-induced gastric injury, where oxidative stress and inflammation are closely interconnected [52]. Although suppression of MAPK/NF-κB-associated signaling was observed following SPE treatment, these pathways represent generalized stress-responsive inflammatory networks. Therefore, the present findings should be interpreted as evidence of pathway-associated modulation rather than direct mechanistic inhibition of specific signaling proteins. Therefore, SPE may represent a promising natural therapeutic candidate for the prevention and management of gastric mucosal injury.

## 4. Materials and Methods

### 4.1. Plant Material and Preparation of Extract

*Salvia plebeia* was harvested in Cheonan, Chungcheongnam-do, Republic of Korea, between May and June. The collected plant material was naturally air-dried for three days and subsequently pulverized into a fine powder. The dried powder was extracted with 70% ethanol using a reflux extraction system for 2 h and 30 min. The resulting extract was filtered through Whatman filter paper (150 mm) to remove insoluble debris. The filtrate was then concentrated and lyophilized using a freeze dryer (Il-Shin Co., Seoul, Republic of Korea) for 48 h. The obtained dry extract powder was stored at −20 °C until further use in the experiments.

### 4.2. HPLC–PDA Analysis of SPE

To characterize the phytochemical profile of SPE, high-performance liquid chromatography coupled with a photodiode array detector (HPLC–PDA; Waters Corporation, Milford, MA, USA) was performed. SPE samples were dissolved in methanol at a concentration of 10 mg/mL, sonicated for 30 min, and filtered through a 0.22 μm membrane filter prior to injection. Standard solutions of rosmarinic acid, luteolin, and hispidulin were prepared in methanol and used as reference compounds for peak identification. Chromatographic separation was carried out using a reversed-phase C18 column (250 × 4.6 mm, 5 μm; Waters Corporation, Milford, MA, USA). The mobile phase consisted of solvent A (0.1% formic acid in water) and solvent B (acetonitrile containing 0.1% formic acid), using a gradient elution system. The flow rate was set at 1.0 mL/min, and the column temperature was maintained at 30 °C. The injection volume was 10 μL, and detection was performed at 330 nm. Peak identification was achieved by comparing retention times with those of authentic standards. Chromatographic data were analyzed using instrument software, and representative chromatograms were obtained for qualitative assessment of the major constituents.

### 4.3. Analysis of Antioxidant Activity

The antioxidant activity of the extracted samples was analyzed using 2,2-diphenyl-1-picrylhydrazyl (DPPH) and 2,2′-azino-bis (3-ethylbenzothiazoline-6-sulfonic acid) (ABTS). 200 μL of the sample supernatant was mixed with 800 μL of 0.4 mM DPPH solution dissolved in 95% ethanol, left in the dark for 5 min, and the absorbance was measured at 517 nm. Ascorbic acid was used as a control at a concentration of 100 μg/mL. 7 mM ABTS and 2.45 mM potassium persulfate were mixed in a 1:1 (*v*/*v*) ratio and reacted at room temperature in the dark for 16 h to prepare the ABTS^+^ solution. The solution was diluted to an absorbance of 1.5 at 734 nm and used in the experiment. 190 μL of ABTS^+^ solution and 10 μL of the sample supernatant were mixed and left to stand for 30 min, after which the absorbance was measured. 1 mg/mL of trolox was used as a control.

### 4.4. Analysis of Total Polyphenol Content

Total polyphenol content (TPC) was determined by mixing 100 μL of the sample extract and standard substance (gallic acid) with 500 μL of 10% Folin & Ciocalteu’s phenol reagent and reacting at 37 °C for 5 min. Subsequently, 600 μL of 2% sodium carbonate was added, and the mixture was reacted at room temperature for 30 min; afterward, the absorbance was measured at 765 nm. The measured values were expressed as mg gallic acid equivalents (GAE)/g relative to the standard substance.

### 4.5. Cell Culture

RAW 264.7 cell were cultured in DMEM medium containing 10% FBS and penicillin–streptomycin at 37 °C under 5% CO_2_ conditions. Cells were subcultured when they reached approximately 70% density and all experiments used cells that had been subcultured no more than four times under the same conditions.

### 4.6. Western Blot Analysis

RAW 264.7 cells were seeded in 6-well plates at a density of 5 × 10^5^ cells per well. After stabilization, cells were pretreated with the indicated samples at concentrations of 100, and 200 μg/mL for 1 h, followed by stimulation with lipopolysaccharide (LPS; 1 μg/mL) for 24 h. Following treatment, cells were washed with phosphate-buffered saline (PBS), and total proteins were extracted using RIPA lysis buffer. Protein concentrations were determined using a Bradford protein assay kit (Sigma-Aldrich, St. Louis, MO, USA). Equal amounts of protein were separated by 10% SDS–polyacrylamide gel electrophoresis (SDS–PAGE) and subsequently transferred onto polyvinylidene difluoride (PVDF) membranes. The membranes were blocked with 5% skim milk in Tris-buffered saline containing 0.1% Tween-20 (TBS-T) for 1 h at room temperature to prevent nonspecific binding. The membranes were then incubated overnight at 4 °C with primary antibodies against TLR4 (rabbit monoclonal, 1:2000; Cell Signaling Technology, Danvers, MA, USA, #14358), JNK (rabbit monoclonal, 1:2000; Cell Signaling Technology, #9252), IκBα (rabbit monoclonal, 1:2000; Cell Signaling Technology, #4814), and β-actin (mouse monoclonal, 1:5000; Cell Signaling Technology, #3700). After washing with TBS-T, the membranes were incubated with horseradish peroxidase (HRP)-conjugated secondary antibodies (anti-rabbit IgG or anti-mouse IgG, 1:3000) for 1 h at room temperature. Protein bands were visualized using an enhanced chemiluminescence reagent (EzWestLumi Plus, ATTO, Tokyo, Japan) and detected using a ChemiDoc imaging system (LuminoGraph, ATTO). Band intensities were quantified using ImageJ software (version 1.54, NIH, Bethesda, MD, USA)., and protein expression levels were normalized to β-actin.

### 4.7. Experimental Animals and Group

Male Sprague–Dawley (SD) rats (8 weeks old) were purchased from Samtako Bio Korea (Osan, Republic of Korea). Animals were acclimatized for one week prior to experimentation. The average body weight of the rats ranged from 350 to 400 g at the start of the study. Animals were fasted for 24 h prior to experimental procedures, from 09:00 on the day before the experiment to 09:00 on the experimental day, while water was provided ad libitum. Rats were housed under controlled environmental conditions with a 12 h light/dark cycle, ambient temperature of 23 ± 2 °C, and relative humidity of 50 ± 10%. Rats were randomly assigned to five groups using a randomization protocol to ensure unbiased allocation (Table 1): Control (CON; no HCl/ethanol treatment, distilled water), HCl/ethanol control (HEC; 150 mM HCl/60% ethanol with distilled water), positive control (POS; 150 mM HCl/60% ethanol with ranitidine at 30 mg/kg), low-dose treatment (SPC-100; 150 mM HCl/60% ethanol with *Salvia plebeia* extract at 100 mg/kg), and high-dose treatment (SPC-300; 150 mM HCl/60% ethanol with *Salvia plebeia* extract at 300 mg/kg). Each group consisted of five animals (*n* = 5). Ranitidine was included as a reference gastroprotective agent for comparative efficacy evaluation and not as a mechanistic comparator for antioxidant or anti-inflammatory signaling pathways. The Wonkwang University Animal Experimental Ethics Committee approved the animal experiments (approval no. WKU25-85).

### 4.8. Acute Gastritis

Acute gastric injury was induced with minor modifications of a previously described HCl/ethanol model. After 24 h of fasting with free access to water, rats were orally administered distilled water, ranitidine (30 mg/kg), or SPE (100 or 300 mg/kg). One hour after pretreatment, acute gastric mucosal injury was induced by oral administration of 1 mL of HCl/ethanol solution (150 mM HCl in 60% ethanol), except in the normal control group. Clinical symptoms were assessed during the post-challenge period under identical observation conditions. Animals were euthanized at the predetermined endpoint, and gastric tissues and gastric juice were collected for subsequent gross, biochemical, histological, and molecular analyses.

### 4.9. Clinical Symptom Evaluation

Clinical symptoms were assessed after HCl/ethanol administration based on locomotor activity, responsiveness to external stimuli and respiratory patterns. Three independent observers evaluated each animal under identical conditions, and the observations were repeated three times within the defined observation period. The final clinical score for each animal was calculated as the mean of the three measurements. Scores were assigned as follows: 1 point, near-normal locomotion with grooming behavior; 5 points, reduced or sluggish movement in response to stimulation; 10 points, little or no spontaneous movement with weak responsiveness; and 15 points, absence of movement accompanied by decreased respiratory rate or deep breathing patterns (Table 2). All clinical symptom evaluations and histopathological assessments were performed in a blinded manner, and the observers were unaware of the experimental group allocation during scoring.

### 4.10. Evaluation of Gastric Lesions and Inhibition Ratio

At the experimental endpoint after HCl/ethanol challenge, rats were anesthetized and euthanized, and the stomachs were immediately excised. The stomachs were opened along the greater curvature, gently rinsed with saline, and photographed for gross examination. The hemorrhagic lesion area was quantified using Image-Pro Plus 7.0 (Media Cybernetics, Bethesda, MD, USA). The inhibition ratio (%) of gastric lesions was calculated relative to the HCl/ethanol-treated control group using the following formula:

Inhibition ratio (%) = [(lesion area of HEC group − lesion area of treated group)/lesion area of HEC group] × 100.

### 4.11. Measurement of Gastric Juice Volume and pH

Immediately after sacrifice, gastric contents were collected from each stomach. The collected gastric juice was centrifuged at 3000 rpm for 15 min, and the supernatant was used for analysis. Gastric juice volume was measured directly in milliliters, and pH was determined using a calibrated pH meter.

### 4.12. Cytokine Levels

Blood samples were collected at sacrifice, allowed to clot at room temperature, and centrifuged to obtain serum. Serum levels of TNF-α and IL-6 were measured using commercially available ELISA kits (R&D Systems, Minneapolis, MN, USA)according to the manufacturers’ instructions. Briefly, standards and serum samples were added to antibody-coated wells and incubated for the recommended period, followed by sequential washing, incubation with detection conjugate, substrate development, and reaction termination. Absorbance was measured at 450 nm using a microplate reader (SpectraMax M2, Molecular Devices, San Jose, CA, USA). Cytokine concentrations were calculated from standard curves generated for each assay.

### 4.13. Tissue Collection and Protein Extraction

Gastric tissues were harvested from rats in each experimental group for the evaluation of inflammatory signaling protein expression. The collected tissues were immediately homogenized in radioimmunoprecipitation assay (RIPA) buffer (Thermo Fisher Scientific, Waltham, MA, USA) supplemented with protease and phosphatase inhibitor cocktails. The homogenates were incubated on ice for 60 min to ensure complete lysis and subsequently centrifuged at 12,000 rpm for 30 min at 4 °C. The resulting supernatants were carefully collected, and total protein concentrations were determined using a bicinchoninic acid (BCA) protein assay kit (Thermo Fisher Scientific, Waltham, MA, USA) according to the manufacturer’s instructions.

### 4.14. Quantitative Real-Time PCR (qRT-PCR) Analysis

Gastric tissue samples obtained from each experimental group were used for gene expression analysis. Tissues were homogenized, and total RNA was extracted using TRIzol reagent (Invitrogen, Carlsbad, CA, USA) according to the manufacturer’s instructions. The purity and concentration of the isolated RNA were determined by measuring the absorbance ratio (A260/A280), and RNA samples were dissolved in RNase-free water. Complementary DNA (cDNA) was synthesized from total RNA using a reverse transcription kit in accordance with the manufacturer’s protocol. Quantitative real-time PCR was performed using SYBR Green qPCR Master Mix (Biofact, Daejeon, Republic of Korea) and gene-specific primers (Table 3). The amplification of target genes, including iNOS (Nos2) and COX-2 (Ptgs2), was conducted under the following thermal cycling conditions: initial denaturation at 95 °C for 5 min, followed by 40 cycles of denaturation at 95 °C for 10 s and annealing/extension at 60 °C for 40 s. Relative gene expression levels were calculated using the 2^−ΔΔCt^ method with Gapdh as the internal reference gene. All samples were analyzed in triplicate, and amplification specificity was confirmed by melt curve analysis.

### 4.15. Histopathological Analysis

After macroscopic examination of gastric lesions, gastric tissues were fixed in 10% neutral-buffered formalin, embedded in paraffin, sectioned at 4–5 μm thickness, and stained with hematoxylin and eosin (H&E) for histopathological assessment. Gastric mucosal damage was semi-quantitatively evaluated using a modified scoring system based on previously established criteria, focusing on inflammation and atrophic gastritis (Table 4). Individual parameters were scored separately, and the total histopathological score was calculated as the sum of each parameter score. All histological evaluations were performed in a blinded manner to minimize observer bias.

### 4.16. Measurement of Gastric Gland Length

Gastric gland length was assessed using a Nikon Eclipse E200 microscope (Nikon, Tokyo, Japan). Digital images of gastric mucosal sections were analyzed using an Image Measurement System (Focus Technology, Neuenhagen, Germany). For each histological slide, gastric gland length was measured at three randomly selected sites, and the mean value was calculated. The results were expressed as mean ± standard deviation for each experimental group.

### 4.17. Immunohistochemistry (IHC) Analysis

Paraffin-embedded gastric tissue sections were deparaffinized in xylene, rehydrated through a graded ethanol series, and subjected to antigen retrieval using 10 mM citrate buffer (pH 6.0). Endogenous peroxidase activity was blocked using 3% hydrogen peroxide. Furthermore, the sections were incubated overnight at 4 °C with anti-NF-κB p65 antibody (1:100 dilution; Cell Signaling Technology, Danvers, MA, USA, #8242), followed by a biotinylated secondary antibody and streptavidin–HRP. Color development was achieved using the DAB substrate, and the slides were counterstained with hematoxylin [53]. Tissue sections were stained and observed under a bright-field microscope (Olympus BX53, Tokyo, Japan).

### 4.18. Statistical Analysis

Data are presented as mean ± standard deviation (SD). For in vitro assays, values were obtained from three independent experiments. For in vivo experiments, each group consisted of five animals (*n* = 5). Statistical analyses were performed using SPSS software version 12.0 (SPSS Inc., Chicago, IL, USA). Differences among groups were analyzed by one-way analysis of variance (ANOVA) followed by Tukey’s post hoc multiple comparison test. A value of *p* < 0.05 was considered statistically significant. Prior to one-way ANOVA, normality and homogeneity of variance were assessed to confirm the suitability of parametric statistical analysis.

## 5. Conclusions

In this study, *Salvia plebeia* extract (SPE) demonstrated significant gastroprotective effects against HCl/ethanol-induced gastric mucosal injury. SPE exhibited strong antioxidant activity and effectively attenuated inflammatory responses by suppressing MAPK/NF-κB signaling pathways In vitro, SPE modulated the expression of key upstream inflammatory signaling molecules, while in vivo, it reduced gastric lesion severity, pro-inflammatory cytokine levels, and the expression of iNOS and COX-2 in gastric tissues. Furthermore, immunohistochemical analysis demonstrated that SPE attenuated NF-κB p65 immunoreactivity, supporting its role in suppressing inflammatory signaling in gastric tissues. Collectively, these findings suggest that SPE protects against acute gastric injury through combined antioxidant and anti-inflammatory mechanisms, supporting its potential as a therapeutic candidate for the prevention of gastric mucosal damage.

## Figures and Tables

**Table 1:** Experimental design for HCl-ethanol gastritis.

Group	150 mM HCl- 60% Ethanol	Material	Dose	*n*
CON	No	D.W	-	5
HEC	Yes	D.W	-	5
POS	Yes	Ranitidine	30 mg/kg	5
SPC-100	Yes	*Salvia plebeia* extracts	100 mg/kg	5
SPC-300	Yes	*Salvia plebeia* extracts	300 mg/kg	5

**Table 2:** Clinical symptom scores in HCl/ethanol-induced gastritis model rats treated with Salvia plebeia extract (SPE).

Score ^(1)^	*n*	Clinical Symptoms ^(2)^
1 point	5	Showed normal gait and excitable behavior
5 point	5	Moved by stimulation or showed slow movement compared with normal movement
10 point	5	Little or no movement. Slight movement by stimulation
15 point	5	No movement. Decreased respiratory rate and deep breathing

^(1)^ Scores were calculated with the average of three different measurements. ^(2)^ Clinical symptoms were observed by three different persons in each animal for one hour.

**Table 3:** PCR primer sets used in the experiment.

Primer	Sequence (5′→3′)	
iNOS	Forward	AGGCAGAGGTTTGTTGCTTG
	Reverse	CATTGGAAGTGAAGCGTTTC
COX-2	Forward	TGATCTACCCAACAGTCCAC
	Reverse	GCTCCTGCTTGTCTGATAGC
GAPDH	Forward	AGGTCGGTGTGAACGGATTTG
	Reverse	TGTAGACCATGTAGTTGAGGTCA

**Table 4:** Grading criteria for histopathological lesion scores.

Parameter	Score	Criteria
Inflammation	0	No lymphocytic or granulocytic infiltration
	1	Mild mucosal lymphocytic infiltration
	2	Moderate mucosal lymphocytic infiltration, some multifocal mucosal lymphoid aggregates
	3	Extensive multifocal mucosal lymphoid aggregates
	4	Multifocal mucosal and submucosal lymphoid aggregates
Atrophic gastritis	0	Parieral cell and glandular architecture preserved
	1	Minimal parietal cell loss, glandular architecture preserved
	2	Moderate parietal cell loss, glandular architecture preserved
	3	Significant parietal cell loss, glandular branching and hyperplasia
	4	Significant parietal cell loss, glandular branching and hyperplasia with submucosal glandular herniation

## Data Availability

Dataset available on request from the authors.
